# Supplementary material for: The Role of Lymphadenectomy in the Surgical Treatment of Hepatocellular Carcinoma: A Systematic Review and Meta-Analysis
Source: Cancers (Basel). 2024 Dec 13;16(24):4166. doi: 10.3390/cancers16244166 (PMC11674971; doi:10.3390/cancers16244166)
Supplement: Supplementary file 1 [file cancers-16-04166-s001.zip › cancers-3316062-supplementary.pdf]

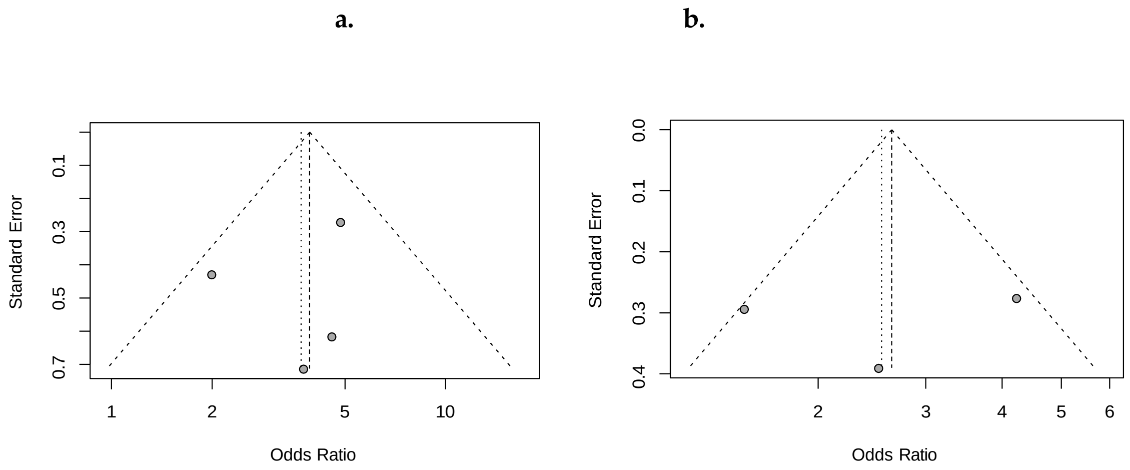

Supplementary Figure S1. Funnel plots.

Supplementary Table S1. Lymph Node Dissection Definition

	**Definition of Lymph Node Dissection (LND)**
**LND, LNM vs no LND**	
Sun H-C, 2007 [18]	Complete regional lymphadenectomy (skeletonization) and/or incisional biopsy
Lee C-W, 2011 [22]	Complete regional lymphadenectomy (skeletonization) and/or incisional biopsy
Hasegawa K, 2014 [24]	Type of lymph node dissection not mentioned; pTNM criteria only
Kemp Bohan PM, 2021 [28]	Type of lymph node dissection not mentioned; pTNM criteria only
**LND, LNM vs LND, LN0**	
Xiaohong S, 2010 [19]	Complete regional lymphadenectomy (skeletonization)
Bergquist JR, 2021 [29]	Excisional biopsy
**LND vs no LND**	
Ravaioli M, 2010 [20]	Complete regional lymphadenectomy (skeletonization)
Wu X, 2015 [25]	Complete regional lymphadenectomy (skeletonization)

Supplementary Table S2. Excluded studies

N°	Reference	Reason for exclusion
**1**	Uenishi, Takahiro, et al. "The clinical significance of lymph node metastases in patients undergoing surgery for hepatocellular carcinoma." Surgery Today 30 (2000): 892-895.	Small sample size
**2**	Bauschke, A., et al. "Which factors affect the long-term survival of patients with hepatocellular carcinoma UICC stage IV?." Journal of cancer research and clinical oncology 142 (2016): 2593-2601.	Small sample size
**3**	Evaluation of the seventh edition of the American Joint Committee on Cancer tumour–node–metastasis (TNM) staging system for patients undergoing curative resection of hepatocellular carcinoma: implications for the development of a refined staging system. Albert C.Y. Chan , Sheung Tat Fan , Ronnie T.P. Poon [1], Tan To Cheung [1], Kenneth S.H. Chok , See Ching Chan, Chung Mau Lo.	No lymphadenectomy vs outcome analysis
**4**	Positive Lymph Node Metastasis Has a Marked Impact on the Long-Term Survival of Patients with Hepatocellular Carcinoma with Extrahepatic Metastasis. Feng Xia ,Lin Wu ,Wan-Yee Lau,Guo Li,Hongbo Huan,Cheng Qian,Kuansheng Ma,Ping Bie. Published: April 23, 2014. https://doi.org/10.1371/journal.pone.0095889.	No lymphadenectomy vs outcome analysis
**5**	Efficacy of Local Treatment in Lymph Node Metastasis from Hepatocellular Carcinoma. Lee B.· Choi J.-Y · Seong J. https://doi.org/10.1159/000529201.	No lymphadenectomy vs outcome analysis
**6**	Surgical treatment of hepatocellular carcinoma: Experience with liver resection and transplantation in 198 patients.Burckhardt Ringe M.D., Rudolf Pichlmayr M.D., Christian Wittekind M.D. & Günter Tusch M.S. https://doi.org/10.1007/BF01659064.	No lymphadenectomy vs outcome analysis
**7**	Metachronous resection of metastatic lymph nodes in patients with hepatocellular carcinoma. Masaji Hashimoto [1], Masamichi Matsuda, Goro Watanabe. PMID: 19621703.	No lymphadenectomy vs outcome analysis
